# The Impact of the Ionic Cross-Linking Mode on the Physical and In Vitro Dexamethasone Release Properties of Chitosan/Hydroxyapatite Beads

**DOI:** 10.3390/molecules24244510

**Published:** 2019-12-10

**Authors:** Maria Jucélia L. Dantas, Bárbara Fernanda F. dos Santos, Albaniza A. Tavares, Matheus A. Maciel, Breno de Medeiros Lucena, Marcus Vinícius L. Fook, Suédina Maria de L. Silva

**Affiliations:** 1 Postgraduate Program in Materials Science and Engineering, Department of Materials Engineering, Federal University of Campina Grande, Campina Grande, PB 58429-900, Brazil; jucelia.lima@hotmail.com (M.J.L.D.); nandasantos_babi@hotmail.com (B.F.F.d.S.); albaniza.alves@gmail.com (A.A.T.); brenolucena.bl@gmail.com (B.d.M.L.); 2 Department of Materials Engineering, Federal University of Campina Grande, Campina Grande, PB 58429-900, Brazil; matheus_alexo@hotmail.com (M.A.M.); marcus.liafook@certbio.ufcg.edu.br (M.V.L.F.)

**Keywords:** beads, chitosan, hydroxyapatite, dexamethasone, ionic cross-linking

## Abstract

In this study, the effect of the ionic cross-linking mode on the ability to control physical properties and in vitro release behavior of the dexamethasone (DEX) drug from chitosan (CS) and chitosan/hydroxyapatite (CS/HA) beads was investigated. CS solutions without and with HA and DEX were dripped into two coagulation solutions, prepared with a non-toxic ionic crosslinker (sodium tripolyphosphate, TPP) and distilled water, one at pH = 9.0 and other at pH = 6.0. Optical microscopy (OM) and scanning electron microscopy (SEM) results showed changes on the surface topology of the beads, with a reduction of roughness for beads prepared at pH = 6.0 and an increase for the one prepared at pH = 9.0. The diameter and sphericity of the beads prepared at pH = 6.0 proved more uniform and had a larger pore size with a good interconnectivity framework. Attenuated total reflectance-Fourier transform infrared spectroscopy (ATR-FTIR) suggested a higher crosslinking degree for beads prepared at pH = 6.0, corroborated by X-ray diffraction profiles (XRD) analysis that indicated a decrease in the crystalline structure for such beads. In in vitro drug release data, all beads presented a sustained release during the studied period (24 h). The drug release rate was affected by the pH of the coagulation solution used in the preparation of the beads. The in vitro kinetics of the release process was of the Peppas–Sahlin model, controlled by both diffusion and relaxation of polymer chains or swelling (anomalous transport mechanism). Our results suggest that DEX-loaded CS/HA beads, crosslinked in TPP coagulation solution at pH = 9.0, led to a decrease in the DEX release rate and prolonged the release period. Thus, this composition might have prospective as a functional material for bone and cartilage tissue engineering.

## 1. Introduction

Polymeric material suitable for biomedical applications must be biocompatible and biodegradable [1]. Due to its biodegradability generating non-toxic degradation products, nonimmunogenic, noncarcinogenic, cytocompatibility, biodegradable, and commercial availability, chitosan (CS) has been gaining increasing importance in tissue engineering, wound healing, and drug delivery [2]. In addition, the cytocompatibility of CS due to its chemical structure, which has a similar backbone to glycosaminoglycan, the major component of the extracellular matrix of bone, CS has been suggested as a natural polymer for use in orthopedic applications and has been widely used for bone [3].

Chitosan can be modified in different ways to control the rate of release and efficiency of the bioactive agent in the delivery systems, for example, from cross-linked beads [4]. As it has an amino group, chitosan can be anionically cross-linked, for example, with sodium tripolyphosphate (TPP), which is a non-toxic reagent, to obtain rigid structures with greater mechanical strength and release control of encapsulated growth factors [5].

The practice of the beads-based delivery system allows control of the drug release profile and the specific target site by carefully adapting the formulation of various drug combinations and polymeric materials. This type of delivery system provides longer life, controlled release rate, and in addition, directs the specific drug. There are different methods for forming beads, for example, interaction with counterions, such as anions (sulfate, phosphates, hydroxides), cross-linking, solvent evaporation, ionic gelling, spray drying, emulsion polymerization, and precipitation, etc. [6].

To improve the properties and increase the application of chitosan potential, it is common to combine materials with different characteristics in the manufacture of spheres, such as the combination of calcium phosphates (such as hydroxyapatite) with polymers [7]. One of the major biomedical applications of chitosan/hydroxyapatite (CS/HA) composites is their use as scaffolds in bone repair or bone regeneration [3,8]. The microsphere-type scaffolds can provide more versatile applications than pre-shaped scaffolds [9]. For example, they can act as injectable scaffolds because of their pseudoplastic behavior. They are easier to injected into the location of bone defects and more advantageous in filling the irregular bone defect compared with films and 3D scaffolds [3].

Hydroxyapatite (HA) is one of the most commonly used ceramics for tissue engineering because of its close resemblance to the chemical composition of the inorganic part of natural bone, which is formed by calcium phosphate (about 60% by weight), collagen (20–30 wt%) and water (10–20 wt%) [10]. In addition to stimulating osteoconductivity and integrating with natural bone without causing any immune response, it can be applied as a drug delivery carrier, Weerasuriya, et al. [11].

Drug-loaded CS/HA composites hold great promise for improving the therapeutic efficiency in tissue regeneration with the aid of functional drugs. At implantation sites, the potential exists for drugs entrapped within the composites to be delivered in a controlled manner over a period of treatment, thus alleviating the postoperative complications as well as promoting the regeneration of new tissues [12]. In this context, dexamethasone (DEX), a potent synthetic glucocorticoid often used to treat a broad spectrum of autoimmune and inflammatory diseases, has been used as a pharmaceutical component [13,14]. It suppresses the immune response, promotes the metabolism of proteins, fats, and carbohydrates, and stimulates the differentiation of osteogenic cells. Traditional oral routes of DEX administration often lead to serious side effects, such as glucocorticoid-induced osteoporosis [13].

Research on dexamethasone-loaded chitosan scaffolds has been conducted [15,16,17,18]. However, the relationship between chitosan, hydroxyapatite, and dexamethasone as a composite applicable to bone tissue engineering was poorly explored. Gao, et al. [19] prepared injectable hydrogels of chitosan/p-glycerophosphate with hydroxyapatite nanoparticles loaded with DEX, the results revealed that the combination of these materials could promote osteoblastic proliferation and differentiation, showing potential for orthopedic regenerative applications. To our knowledge, systematic studies on spheres using the three materials of interest and the evaluation of their mechanical, physical, and biological properties have not been reported. Therefore, the objective of this study was to prepare chitosan beads with hydroxyapatite loaded with the dexamethasone drug by ionotropic gelation method and to evaluate the interactions of materials and possible differences related to pH, to be used as a basis for the manufacture of scaffolds in biomedical applications, including controlled drug release systems for bone regeneration.

## 2. Results and Discussion

### 2.1. Beads Morphology

**Figure 1:**
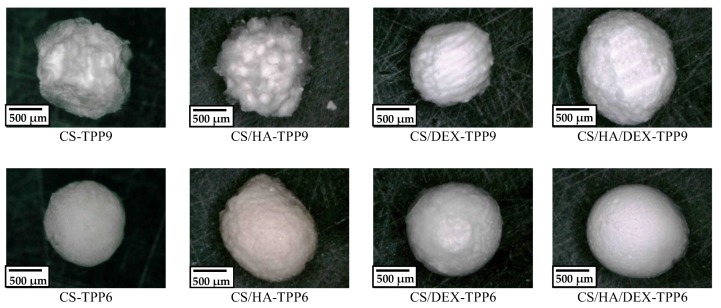
Optical microscopy of the chitosan (CS), chitosan/hydroxyapatite (CS/HA), chitosan/dexamethasone (CS/DEX), and CS/HA/DEX beads prepared in coagulant solution of sodium tripolyphosphate pH = 9.0 (TPP9) and sodium tripolyphosphate pH = 6.0 (TPP6).

Optical microscopy studies revealed that the pH of TPP solution (coagulation solution) used to fabricate CS beads was found to affect their morphology (Figure 1).

Beads prepared in coagulation solution at pH = 6.0 (CS-TPP6, CS/HA-TPP6, CS/DEX-TPP6, and CS/HA/DEX-TPP6) had a smooth surface. On the other hand, surface roughness was visible on beads prepared at pH = 9.0 (CS-TPP9, CS/HA-TPP9, CS/DEX-TPP9, and CS/HA/DEX-TPP9). This could be attributed to the higher ionic crosslink density within the beads fabricated in the coagulation solution at pH = 6.0. This is consistent with previous reports [20,21,22,23]. In addition, the diameter and sphericity of the beads prepared in coagulation solution at pH = 6.0 (Table 1) have not changed statistically with the incorporation of HA, DEX, and the blend HA/DEX. These data were evaluated using analysis of variance (ANOVA), and the significance of the model was verified with the F test. In the significant models, the averages were compared with Tukey’s test, with a significance level of 95% (*p* < 0.05) using Sisvar 5.6.

**Figure 2:**
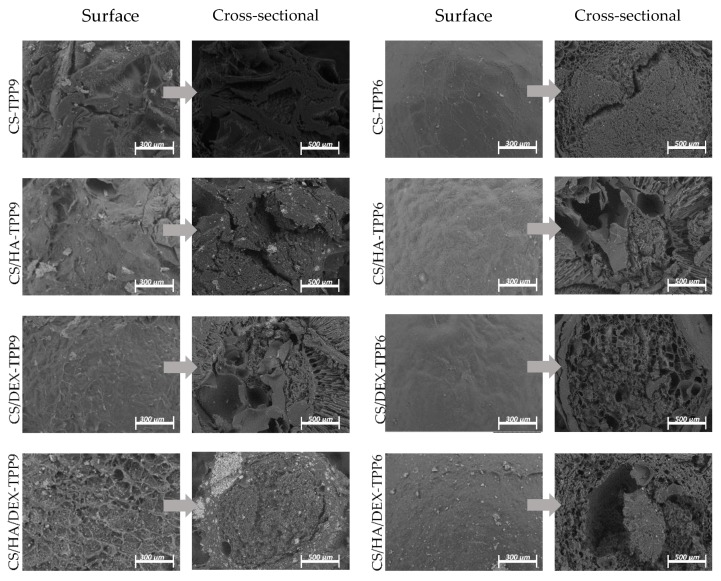
Surface and cross-sectional SEM images of CS, CS/HA, CS/DEX, and CS/HA/DEX cross-linked in TPP9 (**left**) and TPP6 (**right**).

Surface and cross-sectional SEM images of CS, CS/HA, CS/DEX, and CS/HA/DEX beads crosslinked in coagulation solution at pH = 9.0 (TPP9) and pH = 6.0 (TPP6), followed by freezing and lyophilization, using the ice particulates as the porogen materials, are shown in Figure 2. All beads crosslinked at TPP9 had a rougher surface, whereas the ones crosslinked at TPP6 seemed to be significantly densified with negligible pores, corroborating with the results of optical microscopy presented in Figure 1.

Analyses of cross-sections of the beads showed a pore structure where pore size, size distribution, and interconnectivity were affected by coagulation solution pH (Figure 2). The beads prepared in coagulation solution at pH = 6.0 (CS-TPP6, CS/HA-TPP6, CS/DEX-TPP6, and CS/HA/DEX-TPP6) showed a structure with more open pores and more uniformly distributed, i.e., with a better interconnectivity framework, especially the ones prepared with DEX (CS/DEX-TPP6) and HA/DEX (CS/HA/DEX-TPP6). Although pore sizes were not measured in this study, it could be inferred that they were smaller for the beads prepared with HA (CS/HA/DEX-TPP6 and CS/HA/DEX –TPP9). This morphology should be suitable as controlled drug delivery devices and could make access to fluids more difficult and retarding both the diffusion process and release rate of drugs. In addition, it could also allow cell penetration and migration, tissue in-growth and vascularization, and nutrient supply within the bone graft, shortening the healing time, and enhance clinical effect [24,25,26]. Furthermore, the existence of the HA phase in the beads will improve the biomineralization and bioactivity [27,28]. The decrease in the porosity with HA addition to chitosan may be because the HA occupied more space in ice in the frozen mixture resulting in the lower porosity. This is in accordance with previously reported literature [25,29,30,31,32,33].

**Figure 3:**
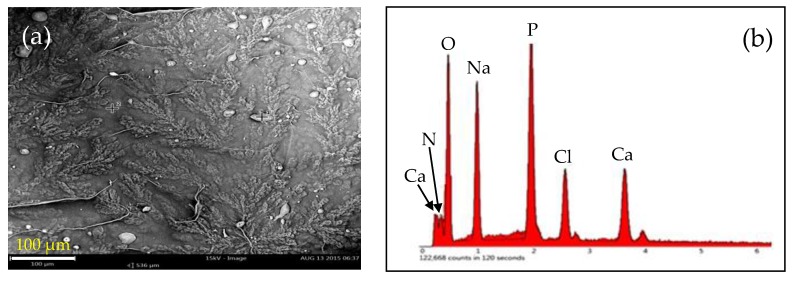
SEM image (**a**) and energy dispersive spectrum (EDS) (**b**) of CS/HA/TPP6 indicating the peaks of nitrogen (N), sodium (Na), phosphorus (P), chlorine (Cl), and calcium (Ca).

Finally, HA particles were scattered in the CS bead uniformly (Figure 3a). The presence of the elements, calcium (Ca), phosphorus (P), nitrogen (N) proper of calcium phosphates and the element sodium (Na) and also P typical of crosslinking of the beads with TPP, and the presence of chlorine (Cl) and sodium (Na) peaks that are perhaps related to the residues of the PBS solution used to washing beads (Figure 3b).

### 2.2. ATR-FTIR Analysis

**Figure 4:**
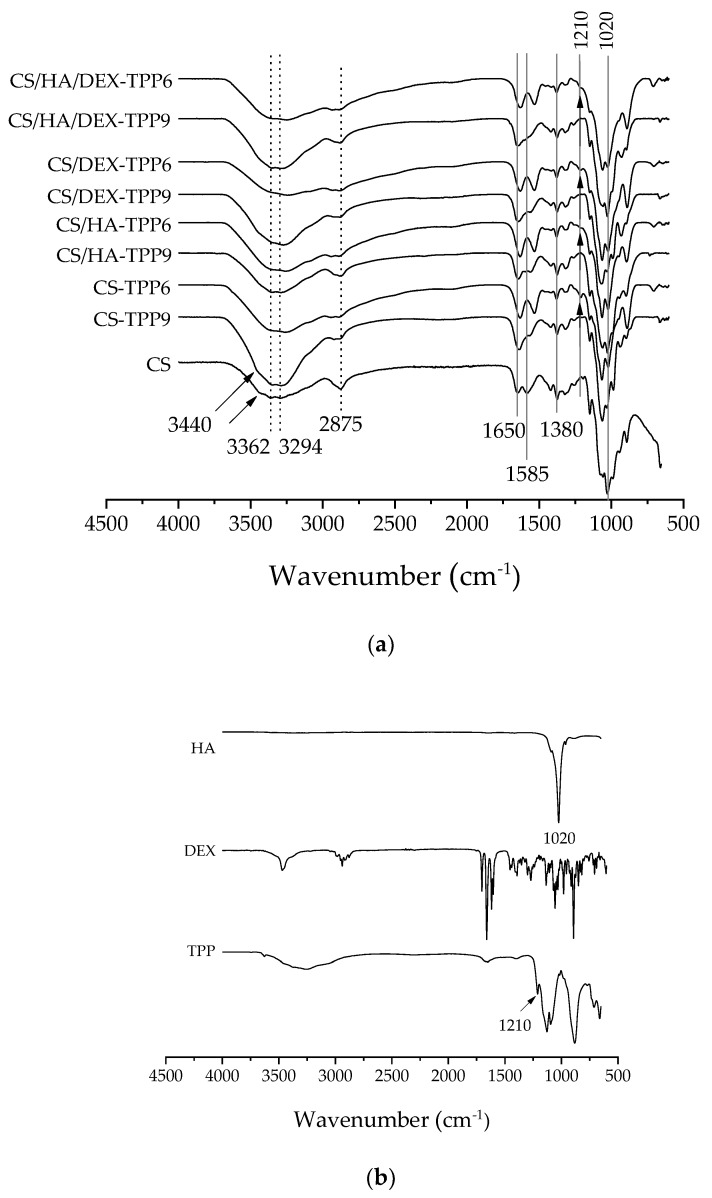
FTIR spectra of (**a**) non-crosslinked CS powder, crosslinked CS beads in coagulation solution at pH = 9.0 (CS-TPP9, CS/HA-TPP9, CS/DEX-TPP9, CS/HA/DEX-TPP9) and pH = 6.0 (CS-TPP6, CS/HA-TPP6, CS/DEX-TPP6, CS/HA/DEX-TPP6), and (**b**) raw materials (TPP, DEX, HA).

Attenuated total reflectance-Fourier transform infrared spectroscopy (ATR-FTIR) spectra of non-crosslinked chitosan (CS) powder and crosslinked CS beads at coagulation solution pH = 9.0 (CS-TPP9, CS/HA-TPP9, CS/DEX-TPP9, CS/HA/DEX-TPP9) and pH = 6.0 (CS-TPP6, CS/HA-TPP6, CS/DEX-TPP6, CS/HA/DEX-TPP6) are shown in Figure 4, along with the spectra of the raw materials separately (TPP, DEX, and HA). These analyses were conducted to evaluate the influence of reaction conditions (pH of coagulation solution) on the chemical structure of chitosan beads.

After the CS crosslinking process, spectral changes were detected (Figure 4a). The first spectral changes observed were in the 3600–2700 cm^−1^ region, where the OH stretching (3440 cm^−1^) and NH_2_ symmetric stretching (3362 cm^−1^) and NH_2_ asymmetric stretching (3294 cm^−1^) absorption bands involved in hydrogen bonds became less prominent and broadened with crosslinking process, especially for CS beads crosslinked in TPP solution at pH = 6.0 (CS-TTP6, CS/HA-TPP6, CS/DEX-TPP6, and CS/HA/DEX-TPP6). Likewise, a broadening and decrease in the intensity of the CH asymmetric stretching vibration peak (2875 cm^−1^) were observed. The reason for these variations is the break of some hydrogen bonds in the CS structure during the crosslinking process and the establishment of new H-bonds between CS and TPP [34]. Moreover, the band at 1650 cm^−1^ (C=O stretching in amide group, –NHCOCH_3_ due to the partial deacetylation of chitosan, amide I vibration) shifted to 1640 cm^−1^ (CS-TPP9) and 1630 cm^−1^ (CS-TPP6). The band at 1585 cm^−1^ (N-H bending in primary amine groups, –NH_2_, overlapping the amide II vibration) shifted to 1556 cm^−1^ (CS-TPP9) and 1534 cm^−1^ (CS-TPP6). The same shifts were observed for the beads prepared with HA, DEX, and HA/DEX (CS/HA-TPP9, CS/DEX-TPP9, CS/HA/DEX-TPP9, CS/HA-TPP6, CS/DEX-TPP6, and CS/HA/DEX-TPP6). For all beads crosslinked at TPP9, the band at 1585 cm^−1^ changed for a broad shoulder at 1556 cm^−1^.

**Figure 5:**
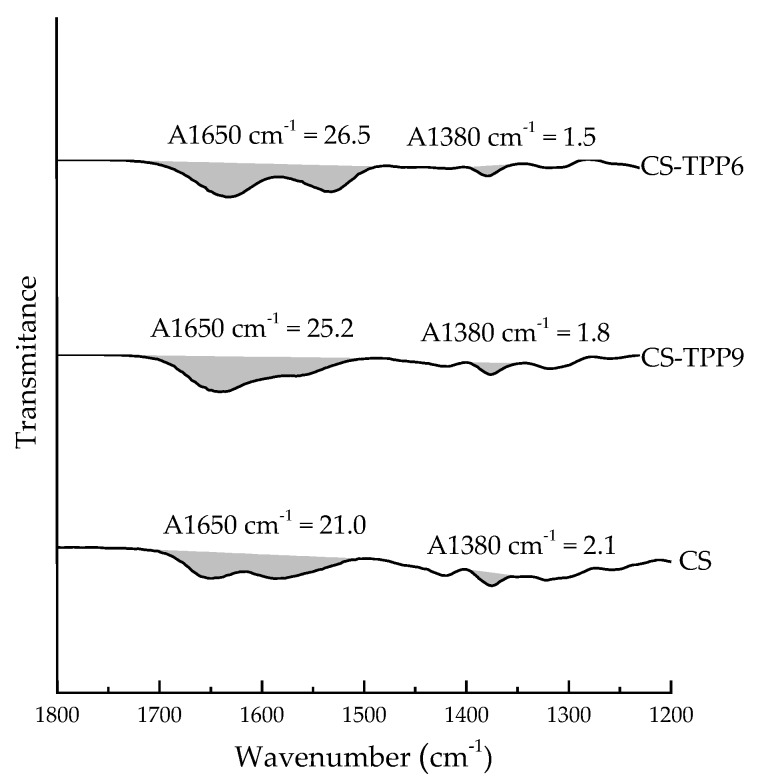
FTIR spectra of CS powder and CS beads crosslinked in coagulation solution at pH = 9.0 (CS-TPP9) and pH = 6.0 (CS-TPP6) showing the baselines for measuring peak area (A1650/A1380) to determine the degree of chitosan protonation.

The shift and intensity of the bands were altered by reaction conditions. The shift and intensity of the bands were higher for the CS beads crosslinked in the TPP solution at pH = 6.0 (TTP6) in comparison with the ones prepared at pH = 9.0 (TPP9) (Figure 4a). The reason may be attributed to the degree of protonation that was higher for CS-TPP6 than for CS-TPP9, ~18 and 14, respectively, as estimated by comparing the ratio of the peak area at 1650 cm^−1^ (NH_3_^+^ vibrational band) to the peak area at 1380 cm^−1^ (which was unaffected by the crosslinking process) (A1650/A1380) (Figure 5) [35]. Thus, for CS crosslinked in TPP solution at pH = 6.0, tripolyphosphate and H^+^ ions predominate and react with amino protonated groups (-NH_3_^+^) chitosan mainly by ionic crosslinking. On the other hand, CS crosslinking in the TPP solution of pH = 9.0, a smaller number of chitosan amino groups was protonated. In this solution, tripolyphosphate and OH^−^ ions were present, and they could competitively react with the -NH_3_^+^ of chitosan by ionic crosslinking and deprotonation, respectively [22,23,36,37,38]. Therefore, the ionic crosslinking density of chitosan beads prepared at pH = 6.0 can be higher than at pH = 9.0. Other researchers [23,39,40,41,42,43,44,45] observed similar results.

The spectra of chitosan beads prepared at pH = 6.0 (CS-TPP6, CS/HA-TPP6, CS/DEX-TPP6, and CS/HA/DEX-TPP6) also exhibited a new band at 1210 cm^−1^ (Figure 4a), consistent with the literature [46,47,48]. This suggests that chitosan might bind with TPP ions more easily at lower pH conditions (pH = 6.0). Hence, the above mentioned changes observed in the FTIR spectra of chitosan after reaction with crosslinking agent TPP confirm that pH of coagulation solution affected the chemical structure of chitosan beads in agreement with the literature [23,39,43].

Finally, the spectra of CS/HA, CS/DEX, and CS/HA/DEX beads exhibited characteristic peaks typically present in chitosan (Figure 4a). The effect of HA and DEX characteristic bands on the beads could be distinguished clearly on CS/HA, CS/DEX, and CS/HA/DEX beads spectra, since the typical ATR-FTIR bands of HA and DEX were overlapping with those of CS. Even so, the peak at 1020 cm^−1^ corresponded to the P–O bond in the HA bulk spectrum (Figure 4b) could be observed on CS/HA spectra (Figure 4a), indicating the presence of HA in the CS beads prepared. In addition, characteristic peaks at the 3600–2700 cm^−1^ region for CS/DEX beads were broadened, suggesting an increase in the hydrogen bonds between DEX drug and CS polymeric chains corroborate with the literature [5,49].

The assignments and corresponding band position for CS, TPP, HA, and DEX are described in Table 2 and are in agreement with the literature [10,42,43,50,51,52,53,54].

### 2.3. XRD Analysis

**Figure 6:**
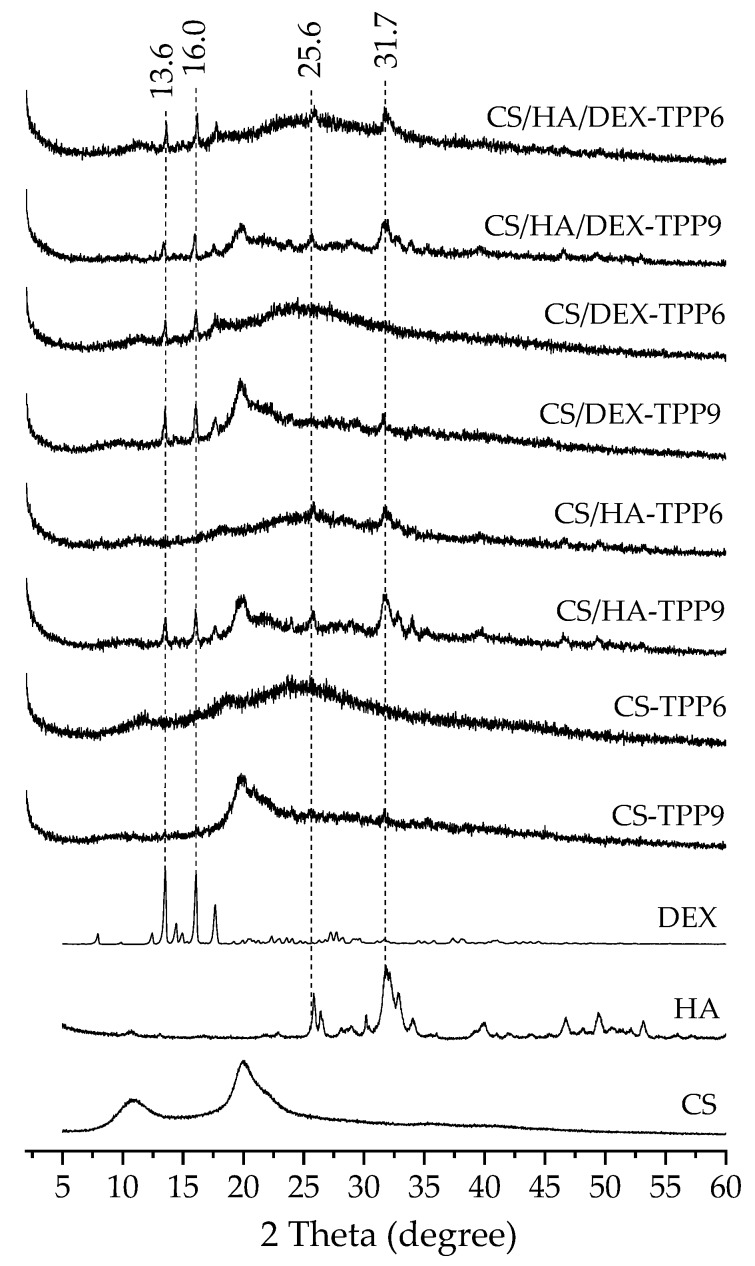
X-ray diffractograms of raw materials (CS, HA, and DEX) and crosslinked CS beads in coagulation solution at pH = 9.0 (CS-TPP9, CS/HA-TPP9, CS/DEX-TPP9, CS/HA/DEX-TPP9) and at pH = 6.0 (CS-TPP6, CS/HA-TPP6, CS/DEX-TPP6, CS/HA/DEX-TPP6).

The X-ray diffraction patterns of non-crosslinked chitosan (CS) and crosslinked CS beads at coagulation solution pH = 9.0 (CS-TPP9, CS/HA-TPP9, CS/DEX-TPP9, CS/HA/DEX-TPP9) and pH = 6.0 (CS-TPP6, CS/HA-TPP6, CS/DEX-TPP6, CS/HA/DEX-TPP6) were compared (Figure 6) to characterize the influence of reaction conditions (pH of coagulation solution) on morphology of the beads.

The diffraction pattern of CS, as shown in Figure 6, exhibited two major crystalline peaks at 10.8° and 20.0°, which confirms the partial crystallinity of the polymer, in agreement with previously reported results [55,56,57,58]. These peaks (10.8° and 20.0°) are typical fingerprints of chitosan related to hydrated and anhydrous crystals, respectively [56]. After crosslinking process, the intensity of these peaks in the XRD patterns significantly decreased (Figure 6), mainly for the beads crosslinked at coagulation solution pH = 6.0 (CS-TPP6, CS/HA-TPP6, CS/DEX-TPP6, CS/HA/DEX-TPP6). The peak at 10.8°, besides presenting lower intensity also presented a little shift in the diffraction angles as shown in Figure 6. The peak at 20.0°, assigned to chitosan chains aligned through intermolecular interactions, became wider and weaker for the beads crosslinked at TPP6 (Figure 6), which corresponds to so-called amorphous halo [23,43]. These results indicated that crystallinity of the chitosan decreased after its crosslinking with TPP especially for the beads crosslinked at coagulation solution pH = 6.0 (TPP6). This is because the CS beads crosslinked at pH = 6.0 (TPP6) were completely ionic-crosslinking dominated and those beads crosslinked at pH = 9.0 (TPP9) were dominated by deprotonation accompanied with slightly ionic crosslinking, corroborating ATR-FTIR data, in agreement with other reports in the literature [35,37,45].

After the incorporation of HA into CS and CS/DEX beads (CS/HA-TPP9, CS/HA-TPP6, CS/HA/DEX-TPP9, and CS/HA/DEX-TPP6), the two main peaks at 2θ value of 25.6° and 31.7° which are (002) and (211) reflections of HA, respectively [59,60,61,62], can be clearly seen in Figure 6. It can also be seen that the broad peak assigned to chitosan at 20.0° becomes wider and weaker with the incorporation of HA. It suggests that the addition of HA obviously affects the crystallinity of chitosan, resulting in a more amorphous structure, possibly due to reduced interactions between chitosan molecules by the presence of HA. Other researchers observed the same behavior [63,64,65,66].

XRD spectra of the CS/DEX-TPP9, CS/DEX-TPP6, CS/HA/DEX-TPP9, and CS/HA/DEX-TPP6 beads (Figure 6), show peaks at 2θ value of 13.6° and 16.0°, which are reflections of dexamethasone (DEX), confirming the presence of DEX crystalline particles at these beads. The incorporation of DEX into CS and CS/HA beads has not affected the crystallinity of chitosan.

### 2.4. In Vitro Drug Release

**Figure 7:**
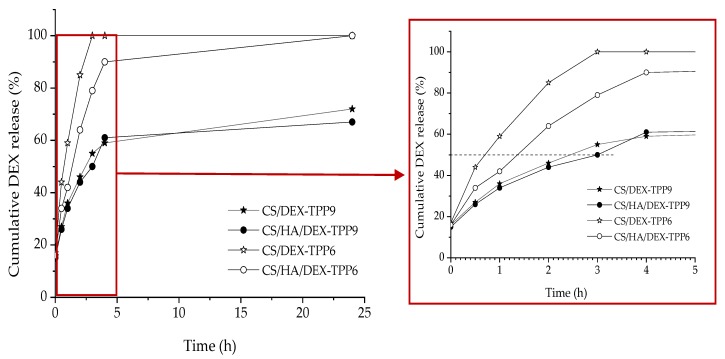
Cumulative percent release of dexamethasone, in PBS pH 7.4 at 37 °C, from the CS and CS/HA beads prepared at coagulation solution pH = 9.0 (CS/DEX-TPP9 and CS/HA/DEX-TPP9) and pH = 6.0 (CS/DEX-TPP6 and CS/HA/DEX-TPP6).

The in vitro release behavior of dexamethasone (DEX) from the CS and CS/HA beads prepared at coagulation solution pH = 9.0 (TPP9) and pH = 6.0 (TPP6) was investigated through UV-visible spectroscopy in PBS up to 24 h at pH 7.4 and 37 °C (Figure 7). According to release profiles, all beads presented a sustained release during the studied period (24 h), but the drug release rate was affected by the pH of the coagulation solution used in the preparation of the beads. The beads prepared at pH = 6.0 (CS/DEX-TPP6 and CS/HA/DEX-TPP6) showed a faster release than the one prepared at pH = 9.0 (CS/DEX-TPP9 and CS/HA/DEX-TPP9). Around 50% of DEX drug was released from the CS/DEX-TPP6 and CS/HA/DEX-TPP6 beads in 42 min and 1 h 36 min, respectively, whereas the same amount of drug was released from the CS/DEX-TPP9 and CS/HA/DEX-TPP9 beads in 2 h 40 min and 3 h, respectively (Figure 7). Beads of CS/DEX-TPP6 and CS/HA/DEX-TPP6 released 100% of the drug within 24 h, whereas about 72% and 67% of the loaded drug from CS/DEX-TPP9 and CS/HA/DEX-TPP9 were released, respectively, within this time. Since the beads prepared at TPP9 exhibits higher crystalline and smaller pore size, according to XRD and SEM data, compared to those prepared at TPP6, the access to release medium was more difficult; thus, both the diffusion process and release rate of drugs were retarded [67].

**Scheme 1:**
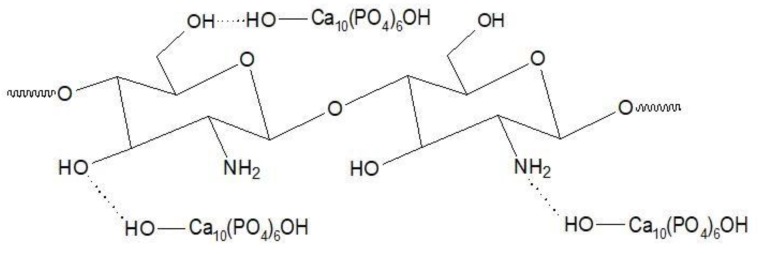
Hydrogen bonds between chitosan and hydroxyapatite [63].

Figure 7 also shows that the incorporation of HA in the CS beads led to a decrease in the DEX release rate and prolonging the release period. The addition of HA in CS indeed reduced the water absorption due to the reduction of polar hydroxyl groups and amino groups in CS/HA beads [68]. As shown in Scheme 1, hydroxyl ions on the surface of HA, Ca_10_(PO_4_)_6_(OH)_2_, might interact with the amino and hydroxyl ions of chitosan by the formation of hydrogen bonds [63,69]. This is in agreement with the XRD data (Figure 6), where it was observed a decrease in chitosan crystallinity with the incorporation of HA, suggesting a hydrogen-bonding interaction between chitosan and hydroxyapatite. These interactions result in the increased physical crosslinking density, and subsequently, due to the decrease in the pore sizes of beads (Figure 2), the water absorption of CS/HA beds is decreased. Therefore, the DEX was strongly kept in the network of the CS/HA beads, causing a decrement in the diffusion of the drug through the beads. Mahdavinia et al. [69] and Asadian-Ardakani, et al. [70] reported identical data for another drug.

### 2.5. Kinetics of Release

To analyze the release mechanism of the dexamethasone (DEX) from chitosan (CS) and the chitosan/hydroxyapatite (CS/HA) beads, prepared at two coagulation solutions (pH = 9.0 and pH = 6.0), over release behavior, the experimental drug release data were fitted to three kinetic models: zero-order, Higuchi, and Peppas–Sahlin [71,72,73,74]. DDSolver (Software Version, Company, City, Country) [75], an add-in program for modeling and comparison of drugs, was used for the analyses. The criteria for selecting the most appropriate model were based on the best goodness of fit of the experimental results, which is based on the statistically higher values of the adjusted coefficient of determination (r^2^), the lower value of Akaike information criterion (AIC), and the largest value of model selection criterion (MSC) [75].

**Figure 8:**
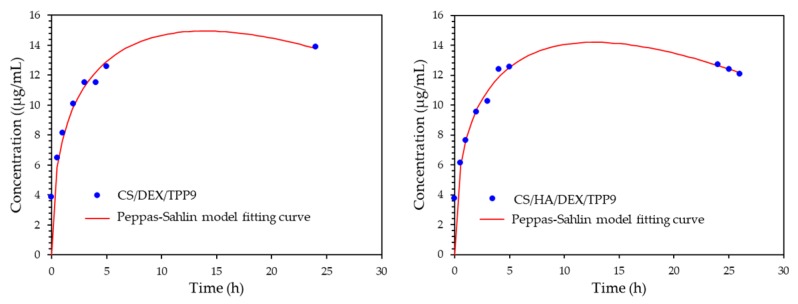

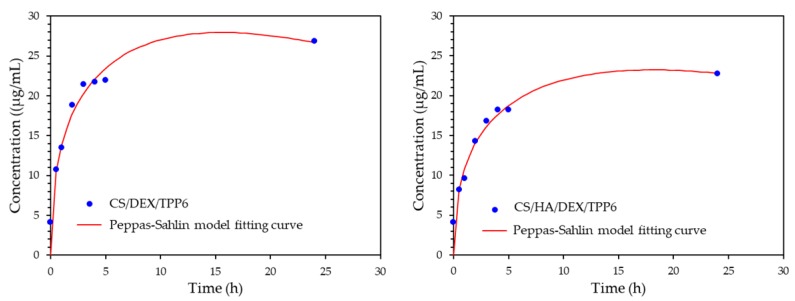
Release kinetics of dexamethasone (DEX), in PBS pH 7.4 at 37 °C, from CS and CS/HA beads prepared at coagulation solution pH = 9.0 (CS/DEX-TPP9 and CS/HA/DEX-TPP9) and pH= 6.0 (CS/DEX-TPP6 and CS/HA/DEX-TPP6) and fit of the Peppas–Sahlin model to the experimental release data.

Table 3 summarizes the values obtained for r^2^, AIC, and MSC considering the CS and CS/HA beads crosslinked at pH = 9.0 (CS/DEX-TPP9 and CS/HA/DEX-TPP9) and pH = 6.0 (CS/DEX-TPP6 and CS/HA/DEX-TPP6). The results show that the DEX release from CS and CS/HA beads, prepared at both pH, showed good fitting with Peppas–Sahlin model, as is exhibited in Figure 8, where higher values of r^2^, lower values of AIC, and higher values of MSC were recorded (Table 3). This model is a semi-empirical model that describes the drug release kinetic from hydrophilic polymers and is applicable for different geometric shapes. This kinetic model points to a release mechanism that relies on the effects of Fickian diffusion and relaxation of polymer chains or swelling (anomalous transport mechanism). Thus, neither absolute Fickian diffusion (as a result of pure drug diffusion) nor zero-order (because of polymer chain relaxation) was the predominant mechanism in this case. This can be assumed as a combination of both contributions of polymer relaxation and drug diffusion factors that determine the drug release mechanism [74,76,77]. According to these results, it can be concluded that coagulation solution pH does not show a significant influence in the drug transport mechanism (anomalous in all the cases).

## 3. Materials and Methods

### 3.1. Materials

Chitosan (CS), derived from the exoskeleton of *Lipopenaeus vannamei* shrimp (company, city, state abbrev if USA, country), 80–93% deacetylation degree, and viscosity average molecular weight of 1.7 × 10^5^ Da, kindly donated by Northeastern Biomaterials Evaluation and Development Laboratory—CERTBIO (Campina Grande, PB, Brazil). This chitosan is accredited by the National Institute of Metrology, Quality, and Technology (INMETRO) at the International Organization of Standardization (ISO)/International Electrotechnical Commission (IEC) 17025:2005 and is used for medical applications. Synthetic hydroxyapatite (HA) with particle size <200 nm, sodium tripolyphosphate (TPP, technical grade, 98%), and dexamethasone D1756 (DEX) were purchased from Sigma–Aldrich^®^ (Darmstadt, Germany). Phosphate buffered saline solution (PBS) at pH = 7.4 and acetic acid (A.C.S reagent, >99%) were purchased from Vetec^®^ (Duque de Caxias, RJ, Brazil). Hydrochloric acid (HCl) (98%) was obtained from Nuclear (São Paulo, SP, Brazil) and ethanol (A.C.S reagent, >99%) was obtained from Neon^®^ (São Paulo, SP, Brazil).

### 3.2. Preparation of Chitosan/Hydroxyapatite Beads (CS/HA)

**Figure 9:**
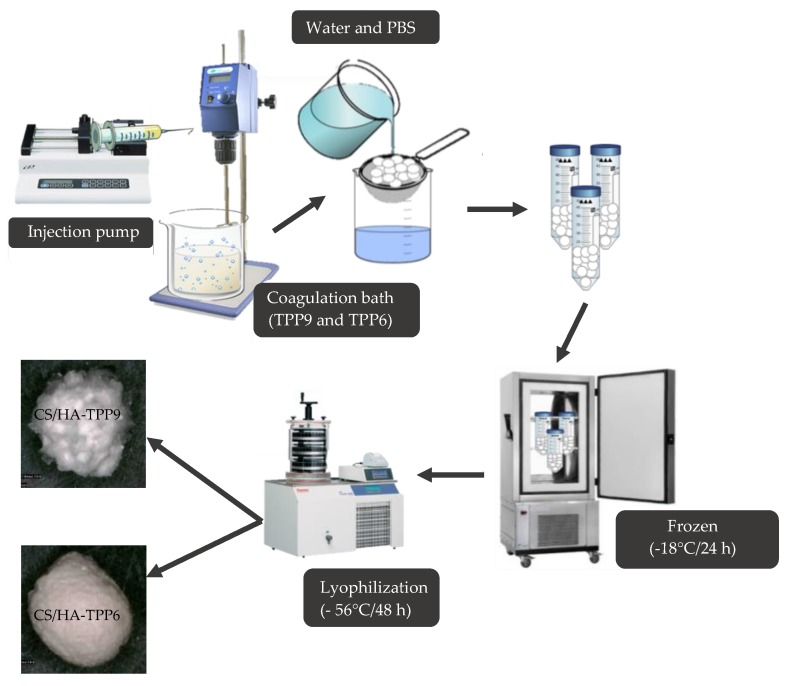
Schematic representation of the production of CS/HA beads by two coagulation baths (TTP9 and TPP6).

Chitosan/hydroxyapatite beads were manufactured by ionotropic gelation followed by lyophilization. First, a 2% (*w*/*v*) chitosan solution was prepared by dissolving chitosan in a 1% (*v*/*v*) aqueous solution of glacial acetic acid. The polymer solution was maintained under constant mechanical stirring (IKA, RW 20, Staufen, Germany) at 75 rpm, at room temperature (25 ± 1 °C) until it became a homogeneous solution. Thereafter, HA powder with a concentration of 20% (*w*/*v*) was added to the chitosan solution, which was maintained under mechanical stirring at 75 rpm until it became a homogeneous mixture. The CS/HA suspension was dripped, using a syringe pump (Pump 11 Pico Plus Elite, Harvard Apparatus, Holliston, MA, USA) and 22G × ½ gauge needles at 25 mL/h, into two coagulation baths (TPP/distilled water solutions). One at pH = 9.0 (coded as TTP9) and other at pH = 6.0 (coded as TPP6) with the same TPP concentration in both baths concentrations (0.1 M). The beads were left in the precipitating solutions for 24 h, at 75 rpm, and 25 ± 1 °C to crosslinking of the beads. Then, the beads were washed with distilled water and PBS solution to a neutral pH (7.4). Afterward, frozen in a freezer (Brastemp clear – 410, São Bernardo dos Campos, SP, Brazil), at approximately −18 °C for 24 h and lyophilized in a lyophilizer (L108, AISI304-LIOTOP stainless steel, São Carlos, SP, Brazil) at −56 °C for 48 h. Subsequently, they were dried at room temperature. The schematic representation of the fabrication of the CS/HA beads through two coagulation baths (TTP9 and TPP6) is shown in Figure 9. CS beads were also fabricated for comparison purposes.

### 3.3. Preparation of Chitosan/Hydroxyapatite/Dexamethasone Beads (CS/HA/DEX)

The CS/HA/DEX beads were prepared by the same method described in Section 3.2. Initially, the drug dexamethasone (DEX) was dissolved in ethanol by dispersing 10% (based on the CS mass) (10 mg mL) under mechanical agitation at 75 rpm at room temperature (25 ± 1 °C). Subsequently, added to the CS/HA solution and maintained under mechanical stirring until homogeneity of the system and the following steps were similar to those presented in Section 3.2.

### 3.4. Characterization of the Beads

#### 3.4.1. Optical Microscopy (OM)

The dimensions of the beads were determined using an optical microscope (Hirox-KH 7700, Tokyo, Japan). About 10 beads were placed on a dry glass slide, positioned at the base of the microscope. The images were made by applying the 5040Z lens and a 100× magnification. The measurements of the beads were carried out using a 5040LOW lens with magnification 20× using the 2D_MEASURE software (2.3.1 Version, Tokyo, Japan). The volumetric diameter (*Dv*), surface diameter (*Ds*), volume (*V*), surface area (*As*), and sphericity (*S*) of the samples were calculated by equations:

(1) DV=(4.Aπ)12

(2) $$D_{S}=\left(\frac{P}{\pi }\right)$$

(3) $$V=\frac{\pi .D_{v}{}^{3}}{6}$$

(4) $$As=\pi .D_{S}{}^{2}$$

(5) $$S=\frac{D_{V}}{D_{S}}$$

where *A* is the area of the particle, and *P* is the perimeter.

#### 3.4.2. Scanning Electron Microscopy (SEM)

The surface and cross-sectional morphology of the beads was observed using scanning electron microscopy (SEM) Phenom-World, Pro-X800-07334 (Eindhoven, The Netherlands). The beads were placed in double carbon tape. The central part of the beads was sectioned with a surgical blade to examine the cross-sectional morphology. SEM images of each bead were taken by applying an electron beam accelerating voltage of 15 kV, with a depth of focus of 1 mm and a resolution of 30 nm. The beads surface deposition of HA was investigated using SEM with energy dispersive spectrum (EDS) (EDAX, Oxford, UK).

#### 3.4.3. Attenuated Total Reflectance-Fourier Transform Infrared Spectroscopy (ATR-FTIR) Analysis

The beads (CS, CS/HA, CS/DEX and CS/HA/DEX), finely grinded using a mortar and pestle, and their pure components (TPP, HA, and DEX) were analyzed by attenuated total reflectance-Fourier transform infrared spectroscopy (ATR-FTIR) using a Perkin Elmer 400 FTIR Spectrometer (Perkin Elmer, MA, USA) equipped with attenuated total reflectance (ATR) accessory employing the Zn-Se crystal. Spectra were recorded over the range of 4000 to 600 cm^−1^ at 4 cm^−1^ resolution, and each spectrum represents 64 co-added scans referenced against an empty ATR cell spectrum.

#### 3.4.4. X-Ray Diffraction (XRD)

X-ray diffraction profiles (XRD) were recorded on a Shimadzu diffractometer model XRD-7000 (Shimadzu, Tokyo/Kyoto, Japan) with Ni-filtered Cu-Kα radiation, a voltage of 40 kV and a current of 30 mA, to identify the composition and crystallinity of the beads (CS, CS/HA, CS/DEX, and CS/HA/DEX), prepared into two coagulation baths (TPP6 and TPP9). The scanning range was from 2θ = 2° to 60° at a scan speed of 0.033°s^−1^. For this characterization, the beads were cryogenically ground and then placed in the sample holder of the diffractometer.

#### 3.4.5. In Vitro Drug Release

The amount of dexamethasone (DEX) released from CS and CS/HA beads was measured using an ultraviolet-visible (UV-VIS) spectrophotometer (Shimadzu, Model 1800, Kyoto, Japan). First, the bead samples (exactly heavy, with masses corresponding to a concentration of 25 µg/mL) were incubated in a volumetric flask in a 50 mL PBS solution (pH = 7.4), as a release medium, and kept under constant stirring of 150 rpm in an IKA shaker model KS4000i (Werke, Germany) at 37 °C ± 0.5 °C to simulate body temperature. Subsequently, an aliquot of the release medium (4 mL) was withdrawn at fixed time intervals (0, 0.5, 1, 2, 3, 4, 5, and 24 h), and replaced with 4 mL of a fresh release medium (at 37 °C ± 0.5 °C) each time. The collected samples were then analyzed for DEX content by measuring the absorbance at 242 nm (λmax of DEX in phosphate buffer solution pH 7.4 measured using a UV-VIS spectrophotometer). All the release experiments were carried out in triplicate, and the average values were used for further data treatment and plotting. The drug concentration was calculated according to a standard curve, and Equation (6) determined the cumulative release. The corresponding drug-release profiles were represented through plots of the cumulative percentage of drug release (calculated from the total amount of DEX contained in each matrix) versus time.

(6) $$\mathrm{Cummulative}\;\mathrm{release}\left(\%\right)=\frac{\sum_{i=0}^{n}C_{i}V_{0}}{m}\times 100$$

where V_0_ is the sampling volume (4.0 mL), Ci is the concentration (mg/L) of the released drug collected at time t, and m is the mass of the drug incorporated in the polymer (1.25 mg).

#### 3.4.6. Release Kinetics

In vitro release data were analyzed by zero-order (Equation (7)), Higuchi (Equation (8)), and Peppas–Sahlin (Equation (9)) kinetic models [71,72,73,74,78,79,80], using the DDSolver [75], an add-in program for modeling and comparison of drugs [81]. This study was carried out to investigate the mechanism of dexamethasone (DEX) release from CS and CS/HA beads, prepared into two coagulation solutions (TPP9 and TPP6). The criteria for selecting the most appropriate model were based on the best goodness of fit of the experimental results, based on the statistically (i) higher values of adjusted coefficient of determination R^2^); (ii) lower value of Akaike information criterion (AIC), and (iii) largest value of model selection criterion (MSC) [75,82].

(7) $$Q_{t}=Q_{0}+k_{0}t$$

where *Q_t_* is the amount of drug released at time *t*; *Q*_0_ the amount of drug in the solution at *t* = 0; (usually, *Q*_0_ = 0) and *k*_0_ the zero-order release constant.

(8) $$Q_{t}=k_{H}t^{1/2}$$

*k_H_* representing the Higuchi rate constant.

(9) $$\frac{Q_{t}}{Q_{\infty }}=k_{1}{\left(t\right)}^{m}+k_{2}{\left(t\right)}^{2m}$$

*Q_∞_* is the maximum amount of the drug released measured at the equilibrium, *k*_1_ and *k*_2_ are the release constants, and *m* is the release exponent.

## 4. Conclusions

Beads of chitosan (CS) and chitosan/hydroxyapatite (CS/HA) loaded with steroid anti-inflammatory agent, dexamethasone (DEX), crosslinked into two sodium tripolyphosphate (TPP) coagulation solutions, one at pH = 9.0 and other at pH = 6.0, with the same concentration of TPP in both solutions (0.1 M), were fabricated by freezing and lyophilization method. The morphology of the beads was influenced by the coagulation solution pH. The beads crosslinked at neutral pH had a rougher surface, whereas the ones crosslinked at acid pH had a smooth surface. The diameter and sphericity of the beads prepared at acid pH proved uniform. Likewise, at this pH, SEM data suggested a larger pore size with a good interconnectivity framework. The crosslinking degree was higher for the beads prepared at acid, as suggested by FTIR-ATR data, corroborating with XRD analysis that indicated a decrease in the crystalline structure for the beads prepared at pH = 6.0. According to in vitro drug release data, all beads presented a sustained release during the studied period (24 h). However, the ones prepared with HA at basic pH (pH = 9.0) led to a decrease in the DEX release rate and prolonging the release period. The in vitro kinetic of DEX from CS and CS/HA beads, prepared at both pH, was best described using the Peppas–Sahlin model. This kinetic model points to a release mechanism that relies on the effects of Fickian diffusion and relaxation of polymer chains or swelling (anomalous transport mechanism). Thus, neither absolute Fickian diffusion (as a result of pure drug diffusion) nor Zero-order (because of polymer chain relaxation) was the predominant mechanism in this case. This can be assumed as a combination of both contributions of polymer relaxation and drug diffusion factors that determine the drug release mechanism. All of these results demonstrate that DEX-loaded CS/HA beads, crosslinked at TPP coagulation solution at basic pH, may be a promising drug carrier for bone tissue engineering.

## Figures and Tables

**Table 1:** Measurements of the beads obtained by optical microscopy.

Sample	Diameter (mm)	Sphericity
CS-TPP9	3.40 A	1.61 A
CS/HA-TPP9	2.78 AB	1.20 BC
CS/DEX-TPP9	2.74ABC	1.32 B
CS/HA/DEX-TPP9	2.07 CD	1.66 A
CS-TPP6	2.06 CD	1.08 C
CS/HA-TPP6	2.49 BCD	1.12 BC
CS/DEX-TPP6	1.96 D	1.09 C
CS/HA/DEX-TPP6	2.38 BCD	1.13 BC

Values followed by the same letter in the column do not differ from each other by the Tukey’s test, considering the nominal value of 5% of significance.

**Table 2:** Infrared band assignments chitosan (CS), sodium tripolyphosphate (TPP), hydroxyapatite (HA), and dexamethasone (DEX).

Sample	Assignment	Wavenumbers (cm^−1^)
Chitosan (CS)	OH stretching	3440
	NH_2_ symmetric stretching	3362
	NH_2_ asymmetric stretching	3294
	−CH asymmetric stretching	2875
	−C=O stretching (amide I)	1650
	−NH_2_ bending (amide I)	1585
	−NH_2_ bending (amide II)	1380
	−C−O asymmetric stretching	1030
Sodium tripolyphosphate (TPP)	P = O stretching	1210
	PO_2_ symmetric stretching	1127
	PO_3_ symmetric stretching	1093
	P–O–P asymmetric bending	881
Hydroxyapatite (HA)	P–O symmetric stretching	961
	P–O asymmetric stretching	1020, 1093
Dexamethasone (DEX)	OH stretching	3400–3675
	−CH symmetric stretching	3020–3055
	−CH asymmetric stretching	2850–3000
	−C=O stretching	1745–1723
	−C=O stretching (carbonyl C_20_)	1700
	−C=O stretching (carbonyl C_3_)	1600
	−C=C stretching	1620
	−C–OH bending	1130

**Table 3:** The DEX loading capacity and release kinetic parameters from CS and CS/HA beads.

Sample	Zero-Order			Higuchi			Peppas–Sahlin		
	r^2^	AIC	MSC	r^2^	AIC	MSC	r^2^	AIC	MSC
CS/DEX-TPP9	0.72	75.35	−1.42	0.90	64.96	−0.13	0.99	52.55	1.42
CS/DEX-TPP6	0.68	59.60	−1.51	0.86	49.70	−0.27	0.99	30.90	2.08
CS/HA/DEX-TPP9	0.64	63.10	−1.82	0.80	55.01	−1.00	0.99	33.21	1.16
CS/HA/DEX-TPP6	0.72	55.72	−1.36	0.90	45.14	−0.04	0.99	29.84	1.87

## References

[1] Banoriya D., Purohit R., Dwivedi R.K. (2017). Advanced application of polymer based biomaterials. Mater. Today Proc..

[2] Zhang X., Zhang Y., Ma G., Yang D., Nie J. (2015). The effect of the prefrozen process on properties of a chitosan/hydroxyapatite/poly (methyl methacrylate) composite prepared by freeze drying method used for bone tissue engineering. Rsc Adv..

[3] Cai B., Zou Q., Zuo Y., Li L., Yang B., Li Y. (2016). Fabrication and cell viability of injectable n-HA/chitosan composite microspheres for bone tissue engineering. Rsc Adv..

[4] Muxika A., Etxabide A., Uranga J., Guerrero P., de la Caba K. (2017). Chitosan as a bioactive polymer: Processing, properties and applications. Int. J. Biol. Macromol..

[5] Uswatta S.P., Okeke I.U., Jayasuriya A.C. (2016). Injectable porous nano-hydroxyapatite/chitosan/tripolyphosphate scaffolds with improved compressive strength for bone regeneration. Mater. Sci. Eng. C.

[6] Ali A., Ahmed S. (2018). A review on chitosan and its nanocomposites in drug delivery. Int. J. Biol. Macromol..

[7] Yu P., Bao R.Y., Shi X.J., Yang W., Yang M.B. (2017). Self-assembled high-strength hydroxyapatite/graphene oxide/chitosan composite hydrogel for bone tissue engineering. Carbohydr. Polym..

[8] Fan T., Chen J., Pan P., Zhang Y., Hu Y., Liu X., Shi X., Zhang Q. (2016). Bioinspired double polysaccharides-based nanohybrid scaffold for bone tissue engineering. Colloids Surf. B Biointerfaces.

[9] Wang Q., Wang J., Lu Q., Detamore M.S., Berkland C. (2010). Injectable PLGA based colloidal gels for zero-order dexamethasone release in cranial defects. Biomaterials.

[10] Nazeer M.A., Yilgör E., Yilgör I. (2017). Intercalated chitosan/hydroxyapatite nanocomposites: Promising materials for bone tissue engineering applications. Carbohydr. Polym..

[11] Weerasuriya D.R.K., Wijesinghe W., Rajapakse R.M.G. (2017). Encapsulation of anticancer drug copper bis(8-hydroxyquinoline) in hydroxyapatite for pH-sensitive targeted delivery and slow release. Mater. Sci. Eng. C.

[12] Liu T.Y., Chen S.Y., Li J.H., Liu D.M. (2006). Study on drug release behaviour of CDHA/chitosan nanocomposites--effect of CDHA nanoparticles. J. Control. Release.

[13] Paun I.A., Zamfirescu M., Luculescu C.R., Acasandrei A.M., Mustaciosu C.C., Mihailescu M., Dinescu M. (2017). Electrically responsive microreservoires for controllable delivery of dexamethasone in bone tissue engineering. Appl. Surf. Sci..

[14] Suryaprakash N. (2014). Sensitivity enhancement in slice-selective NMR experiments through polarization sharing. Chem. Commun..

[15] Tığlı R.S., Akman A.C., Gümüşderelıoğlu M., Nohutçu R.M. (2009). In vitro release of dexamethasone or bFGF from chitosan/hydroxyapatite scaffolds. J. Biomater. Sci. Polym. Ed..

[16] Duarte A.R.C., Mano J.F., Reis R.L. (2009). Preparation of chitosan scaffolds loaded with dexamethasone for tissue engineering applications using supercritical fluid technology. Eur. Polym. J..

[17] Martins A., Duarte A.R., Faria S., Marques A.P., Reis R.L., Neves N.M. (2010). Osteogenic induction of hBMSCs by electrospun scaffolds with dexamethasone release functionality. Biomaterials.

[18] Omidvar N., Ganji F., Eslaminejad M.B. (2016). In vitro osteogenic induction of human marrow-derived mesenchymal stem cells by PCL fibrous scaffolds containing dexamethazone-loaded chitosan microspheres. J. Biomed. Mater. Res. Part A.

[19] Gao C., Cai Y., Kong X., Han G., Yao J. (2013). Development and characterization of injectable chitosan-based hydrogels containing dexamethasone/rhBMP-2 loaded hydroxyapatite nanoparticles. Mater. Lett..

[20] Ogawa K., Sato S., Kokufuta E. (2005). Formation of intra-and interparticle polyelectrolyte complexes between cationic nanogel and strong polyanion. Langmuir.

[21] Huang Y., Lapitsky Y. (2011). Monovalent salt enhances colloidal stability during the formation of chitosan/tripolyphosphate microgels. Langmuir.

[22] Ferreira Tomaz A., Sobral de Carvalho S., Cardoso Barbosa R., L Silva S.M., Sabino Gutierrez M.A., B de Lima A.G., L Fook M.V. (2018). Ionically crosslinked chitosan membranes used as drug carriers for cancer therapy application. Materials.

[23] Bhumkar D.R., Pokharkar V.B. (2006). Studies on effect of pH on cross-linking of chitosan with sodium tripolyphosphate: A technical note. AAPS PharmSciTech.

[24] Saravanan S., Nethala S., Pattnaik S., Tripathi A., Moorthi A., Selvamurugan N. (2011). Preparation, characterization and antimicrobial activity of a bio-composite scaffold containing chitosan/nano-hydroxyapatite/nano-silver for bone tissue engineering. Int. J. Biol. Macromol..

[25] Teng S.H., Lee E.J., Wang P., Jun S.H., Han C.M., Kim H.E. (2009). Functionally gradient chitosan/hydroxyapatite composite scaffolds for controlled drug release. J. Biomed. Mater. Res. Part B Appl. Biomater..

[26] Dhivya S., Saravanan S., Sastry T., Selvamurugan N. (2015). Nanohydroxyapatite-reinforced chitosan composite hydrogel for bone tissue repair in vitro and in vivo. J. Nanobiotechnol..

[27] Kong L., Gao Y., Cao W., Gong Y., Zhao N., Zhang X. (2005). Preparation and characterization of nano-hydroxyapatite/chitosan composite scaffolds. J. Biomed. Mater. Res. Part A.

[28] Zhai Y., Cui F. (2006). Recombinant human-like collagen directed growth of hydroxyapatite nanocrystals. J. Cryst. Growth.

[29] Kim H.W., Knowles J.C., Kim H.E. (2005). Hydroxyapatite and gelatin composite foams processed via novel freeze-drying and crosslinking for use as temporary hard tissue scaffolds. J. Biomed. Mater. Res. Part A.

[30] Jiang L., Li Y., Wang X., Zhang L., Wen J., Gong M. (2008). Preparation and properties of nano-hydroxyapatite/chitosan/carboxymethyl cellulose composite scaffold. Carbohydr. Polym..

[31] Kim H.-L., Jung G.-Y., Yoon J.-H., Han J.-S., Park Y.-J., Kim D.-G., Zhang M., Kim D.-J. (2015). Preparation and characterization of nano-sized hydroxyapatite/alginate/chitosan composite scaffolds for bone tissue engineering. Mater. Sci. Eng. C.

[32] Peter M., Ganesh N., Selvamurugan N., Nair S., Furuike T., Tamura H., Jayakumar R. (2010). Preparation and characterization of chitosan–gelatin/nanohydroxyapatite composite scaffolds for tissue engineering applications. Carbohydr. Polym..

[33] Zheng J.P., Wang C.Z., Wang X.X., Wang H.Y., Zhuang H., De Yao K. (2007). Preparation of biomimetic three-dimensional gelatin/montmorillonite–chitosan scaffold for tissue engineering. React. Funct. Polym..

[34] Pawlak A., Mucha M. (2003). Thermogravimetric and FTIR studies of chitosan blends. Thermochim. Acta.

[35] Cui Z., Xiang Y., Si J., Yang M., Zhang Q., Zhang T. (2008). Ionic interactions between sulfuric acid and chitosan membranes. Carbohydr. Polym..

[36] Tripathy S., Das S., Chakraborty S.P., Sahu S.K., Pramanik P., Roy S. (2012). Synthesis, characterization of chitosan–tripolyphosphate conjugated chloroquine nanoparticle and its in vivo anti-malarial efficacy against rodent parasite: A dose and duration dependent approach. Int. J. Pharm..

[37] Lee S.-T., Mi F.-L., Shen Y.-J., Shyu S.-S. (2001). Equilibrium and kinetic studies of copper (II) ion uptake by chitosan-tripolyphosphate chelating resin. Polymer.

[38] Mi F.L., Shyu S.S., Lee S.T., Wong T.B. (1999). Kinetic study of chitosan-tripolyphosphate complex reaction and acid-resistive properties of the chitosan-tripolyphosphate gel beads prepared by in-liquid curing method. J. Polym. Sci. Part B Polym. Phys..

[39] Mi F.-L., Sung H.-W., Shyu S.-S., Su C.-C., Peng C.-K. (2003). Synthesis and characterization of biodegradable TPP/genipin co-crosslinked chitosan gel beads. Polymer.

[40] Lima H.A., Lia F.M.V., Ramdayal S. (2014). Preparation and characterization of chitosan-insulin-tripolyphosphate membrane for controlled drug release: Effect of cross linking agent. J. Biomater. Nanobiotechnol..

[41] Sacco P., Borgogna M., Travan A., Marsich E., Paoletti S., Asaro F., Grassi M., Donati I. (2014). Polysaccharide-based networks from homogeneous chitosan-tripolyphosphate hydrogels: Synthesis and characterization. Biomacromolecules.

[42] Vimal S., Majeed S.A., Taju G., Nambi K., Raj N.S., Madan N., Farook M., Rajkumar T., Gopinath D., Hameed A.S. (2013). RETRACTED: Chitosan tripolyphosphate (CS/TPP) nanoparticles: Preparation, characterization and application for gene delivery in shrimp. Acta Trop..

[43] Gierszewska M., Ostrowska-Czubenko J. (2016). Chitosan-based membranes with different ionic crosslinking density for pharmaceutical and industrial applications. Carbohydr. Polym..

[44] Mi F.L., Shyu S.S., Kuan C.Y., Lee S.T., Lu K.T., Jang S.F. (1999). Chitosan–polyelectrolyte complexation for the preparation of gel beads and controlled release of anticancer drug. I. Effect of phosphorous polyelectrolyte complex and enzymatic hydrolysis of polymer. J. Appl. Polym. Sci..

[45] Mi F.L., Shyu S.S., Wong T.B., Jang S.F., Lee S.T., Lu K.T. (1999). Chitosan–polyelectrolyte complexation for the preparation of gel beads and controlled release of anticancer drug. II. Effect of pH-dependent ionic crosslinking or interpolymer complex using tripolyphosphate or polyphosphate as reagent. J. Appl. Polym. Sci..

[46] Xu Y., Du Y. (2003). Effect of molecular structure of chitosan on protein delivery properties of chitosan nanoparticles. Int. J. Pharm..

[47] Wang X., Ma J., Wang Y., He B. (2001). Structural characterization of phosphorylated chitosan and their applications as effective additives of calcium phosphate cements. Biomaterials.

[48] Pierog M., Gierszewska-Drużyńska M., Ostrowska-Czubenko J. (2009). Effect of ionic crosslinking agents on swelling behavior of chitosan hydrogel membranes. Prog. Chem. Appl. Chitin Its Deriv. Pol. Chitin Soc. Łódź.

[49] Rodrigues L.B., Leite H.F., Yoshida M.I., Saliba J.B., Junior A.S.C., Faraco A.A. (2009). In vitro release and characterization of chitosan films as dexamethasone carrier. Int. J. Pharm..

[50] Jóźwiak T., Filipkowska U., Szymczyk P., Rodziewicz J., Mielcarek A. (2017). Effect of ionic and covalent crosslinking agents on properties of chitosan beads and sorption effectiveness of Reactive Black 5 dye. React. Funct. Polym..

[51] Shamekhi M.A., Rabiee A., Mirzadeh H., Mahdavi H., Mohebbi-Kalhori D., Eslaminejad M.B. (2017). Fabrication and characterization of hydrothermal cross-linked chitosan porous scaffolds for cartilage tissue engineering applications. Mater. Sci. Eng. C.

[52] Marques J., Chagas J., Fonseca J., Pereira M. (2016). Comparing homogeneous and heterogeneous routes for ionic crosslinking of chitosan membranes. React. Funct. Polym..

[53] Gierszewska-Drużyńska M., Ostrowska-Czubenko J. (2010). The effect of ionic crosslinking on thermal properties of hydrogel chitosan membranes. Prog. Chem. Appl. Chitin Its Deriv..

[54] Rodrigues S., da Costa A.M.R., Grenha A. (2012). Chitosan/carrageenan nanoparticles: Effect of cross-linking with tripolyphosphate and charge ratios. Carbohydr. Polym..

[55] Baskar D., Kumar T.S. (2009). Effect of deacetylation time on the preparation, properties and swelling behavior of chitosan films. Carbohydr. Polym..

[56] Baklagina Y., Klechkovskaya V., Kononova S., Petrova V., Poshina D., Orekhov A., Skorik Y. (2018). Polymorphic modifications of chitosan. Crystallogr. Rep..

[57] Samuels R.J. (1981). Solid state characterization of the structure of chitosan films. J. Polym. Sci. Polym. Phys. Ed..

[58] F dos Santos B.F., Maciel M.A., A Tavares A., QB de Araújo Fernandes C., B de Sousa W.J., Lia Fook M.V., Farias Leite I., de Lima Silva S.M. (2018). Synthesis and preparation of chitosan/clay microspheres: Effect of process parameters and clay type. Materials.

[59] Kamalanathan P., Ramesh S., Bang L., Niakan A., Tan C., Purbolaksono J., Chandran H., Teng W. (2014). Synthesis and sintering of hydroxyapatite derived from eggshells as a calcium precursor. Ceram. Int..

[60] Sumathra M., Sadasivuni K.K., Kumar S.S., Rajan M. (2018). Cisplatin-Loaded graphene oxide/chitosan/hydroxyapatite composite as a promising tool for osteosarcoma-affected bone regeneration. ACS Omega.

[61] Anselme K. (2000). Osteoblast adhesion on biomaterials. Biomaterials.

[62] Chen F., Wang Z.-C., Lin C.-J. (2002). Preparation and characterization of nano-sized hydroxyapatite particles and hydroxyapatite/chitosan nano-composite for use in biomedical materials. Mater. Lett..

[63] Xianmiao C., Yubao L., Yi Z., Li Z., Jidong L., Huanan W. (2009). Properties and in vitro biological evaluation of nano-hydroxyapatite/chitosan membranes for bone guided regeneration. Mater. Sci. Eng. C.

[64] Pallela R., Venkatesan J., Janapala V.R., Kim S.K. (2012). Biophysicochemical evaluation of chitosan-hydroxyapatite-marine sponge collagen composite for bone tissue engineering. J. Biomed. Mater. Res. Part A.

[65] Han J., Zhou Z., Yin R., Yang D., Nie J. (2010). Alginate–chitosan/hydroxyapatite polyelectrolyte complex porous scaffolds: Preparation and characterization. Int. J. Biol. Macromol..

[66] Zhang Y., Venugopal J.R., El-Turki A., Ramakrishna S., Su B., Lim C.T. (2008). Electrospun biomimetic nanocomposite nanofibers of hydroxyapatite/chitosan for bone tissue engineering. Biomaterials.

[67] Martinez A.W., Caves J.M., Ravi S., Li W., Chaikof E.L. (2014). Effects of crosslinking on the mechanical properties, drug release and cytocompatibility of protein polymers. Acta Biomater..

[68] Hu Q., Li B., Wang M., Shen J. (2004). Preparation and characterization of biodegradable chitosan/hydroxyapatite nanocomposite rods via in situ hybridization: A potential material as internal fixation of bone fracture. Biomaterials.

[69] Mahdavinia G.R., Karimi M.H., Soltaniniya M., Massoumi B. (2019). In vitro evaluation of sustained ciprofloxacin release from κ-carrageenan-crosslinked chitosan/hydroxyapatite hydrogel nanocomposites. Int. J. Biol. Macromol..

[70] Asadian-Ardakani V., Saber-Samandari S., Saber-Samandari S. (2016). The effect of hydroxyapatite in biopolymer-based scaffolds on release of naproxen sodium. J. Biomed. Mater. Res. Part A.

[71] Dredán J., Zelkó R., Antal I., Bihari E., Rácz I. (1998). Effect of chemical properties on drug release from hydrophobic matrices. Int. J. Pharm..

[72] Vueba M., De Carvalho L.B., Veiga F., Sousa J., Pina M. (2004). Influence of cellulose ether polymers on ketoprofen release from hydrophilic matrix tablets. Eur. J. Pharm. Biopharm..

[73] Sood A., Panchagnula R. (1998). Drug release evaluation of diltiazem CR preparations. Int. J. Pharm..

[74] Farrag Y., Ide W., Montero B., Rico M., Rodríguez-Llamazares S., Barral L., Bouza R. (2018). Starch films loaded with donut-shaped starch-quercetin microparticles: Characterization and release kinetics. Int. J. Biol. Macromol..

[75] Zhang Y., Huo M., Zhou J., Zou A., Li W., Yao C., Xie S. (2010). DDSolver: An add-in program for modeling and comparison of drug dissolution profiles. AAPS J..

[76] Siepmann J., Peppas N. (2012). Modeling of drug release from delivery systems based on hydroxypropyl methylcellulose (HPMC). Adv. Drug Deliv. Rev..

[77] Ritger P.L., Peppas N.A. (1987). A simple equation for description of solute release I. Fickian and non-fickian release from non-swellable devices in the form of slabs, spheres, cylinders or discs. J. Control. Release.

[78] Higuchi T. (1961). Rate of release of medicaments from ointment bases containing drugs in suspension. J. Pharm. Sci..

[79] Higuchi T. (1963). Mechanism of sustained-action medication. Theoretical analysis of rate of release of solid drugs dispersed in solid matrices. J. Pharm. Sci..

[80] Korsmeyer R.W., Gurny R., Doelker E., Buri P., Peppas N.A. (1983). Mechanisms of solute release from porous hydrophilic polymers. Int. J. Pharm..

[81] da Silva M.C., da Silva H.N., Alves Leal Cruz R.C., Sagoe Amoah S.K., de Lima Silva S.M., Lia Fook M.V. (2019). *N*-acetyl-d-glucosamine-loaded chitosan filaments biodegradable and biocompatible for use as absorbable surgical suture materials. Materials.

[82] Aydin R., Pulat M. (2012). 5-Fluorouracil encapsulated chitosan nanoparticles for pH-stimulated drug delivery: Evaluation of controlled release kinetics. J. Nanomater..

